# Utility of Emergent Spine MRI in the Emergency Department

**DOI:** 10.5811/westjem.32802

**Published:** 2025-07-12

**Authors:** Farid Hajibonabi, Dan Cohen-Addad, Francisco Delgado, Po-Han Chen, Bing Fang Wang, Shamie Das, Tarek N. Hanna

**Affiliations:** * Emory University School of Medicine, Department of Radiology and Imaging Sciences, Atlanta, Georgia; † Agusta University Medical College of Georgia, Department of Radiology, Atlanta, Georgia; ‡ Emory University, Department of Emergency Medicine, Atlanta, Georgia

## Abstract

**Introduction:**

Prolonged emergency department (ED) waiting times for STAT spine magnetic resonance imaging (MRI) in the ED can expose patients to hospital-acquired infections and increase the workload in the ED, further impacting healthcare quality. In this study we aimed to characterize emergent spine MRI frequency and positivity in the ED, and its impact on ED length of stay (LOS), admission rates, and the necessity for surgical interventions.

**Methods:**

We performed a retrospective chart review of a consecutive group of patients who had emergent spine MRI (cervical, thoracic, lumbar) ordered from the EDs at four hospitals from January 1, 2017–December 31,2022 were included for traumatic and atraumatic patients. We recorded patient demographics, time metrics, discharge status, and surgical interventions within seven days (for those who were hospitalized during the ED encounter). Spine MRI reports were reviewed and categorized, with positive cases defined as severe spinal canal stenosis regardless of cause and/or fracture. We used descriptive statistics to assess the positivity rate for emergent spine MRIs as well as the LOS, rate of surgery, and rate of admission for patients getting emergent spine MRIs.

**Results:**

A total of 689 spine MRI of 889,527 ED visits (0.1%) were included. Patients’ mean age was 51.3 ±17.1 years, and 59.5% were female. Discharge rate was 93.9%, 3.3% were admitted, 1.7% left against medical advice, and 1.0% were transferred to other facilities. The overall spine MRI positivity rate was 18.9% (130). Moreover, the median (IQR) time from imaging order placement to imaging completion was 2.6 (1.8 – 3.7) hours, while the time from imaging completion to final report availability was 1.5 (0.4 – 13.9) hours. The median ED LOS was 7.4 (5.7 – 9.5) hours. Of 23 hospitalized patients, 17 (73.9%) required surgical intervention. Positive cases had significantly higher ED LOS compared to negative cases (8.1 vs 7.2, respectively; *P* < .001).

**Conclusion:**

The positivity rate for ED spine MRI in this study was 18.9%. Of the positive cases, 17.7% underwent hospitalization, with 13.1% requiring emergent surgery. Considering high costs in both time and resource utilization, further research is needed to optimize the triage process for patients requiring emergent spine MRI.

## INTRODUCTION

Diagnostic imaging plays a pivotal role in the evaluation of patients presenting with acute spine pathologies in the emergency department (ED). Magnetic resonance imaging (MRI) offers unparalleled visualization of soft-tissue structures, thus allowing for optimal identification of pathologic changes to bone marrow, ligaments, spinal cord, and neural structures.^1^–^7^ Timely and accurate diagnosis of emergent spinal pathologies in the ED is of paramount importance. In trauma, MRI allows for accurate identification and characterization of injury and directs management to improve patient outcomes, which is especially important given the high morbidity and mortality of traumatic spinal cord injuries.^8^,^9^

However, other chronic pathologies, such as degenerative spondylosis, are not considered “emergent” and may not require MRI in the ED setting. For instance, according to the American College of Radiology appropriateness criteria, cervical spine MRI is usually appropriate in the setting of trauma; however, thoracolumbar spine MRI is only appropriate when there are neurologic findings with imaging-confirmed thoracolumbar spine injury.^10^,^11^ In addition, the benefits of MRI must be weighed against its costly and time-intensive nature, particularly in the time-conscious ED setting.^12^ Therefore, while spinal MRI is absolutely appropriate in some cases, the optimal setting—emergent, urgent, or outpatient—for acquiring these images is yet to be determined. Implementation of diagnostic and triage algorithms can decrease unnecessary advanced imaging usage, reduce referrals to spine surgeons, lower healthcare costs, decrease the average time from imaging to diagnosis, and optimize the triage process.^13^–^19^

While some studies have assessed the MRI positivity in trauma patients,^20^ there is limited literature specifically addressing the impact of emergent spine MRI on physician decisions regarding hospitalization or surgery.^21^ Furthermore, additional research is necessary to identify potential areas for improvement in patient triage for emergent spine MRI and to establish a system to meet patients’ need in the ED. In this study we aimed to determine the prevalence of positive spine MRI results overall and across different spinal segments in four urban EDs and to assess the associated admission rates, the number of resulting surgical interventions, and the impact of these MRIs on the length of stay (LOS) in the ED.

## METHODS

### Study Design and Setting

We performed a retrospective chart review of patients from four hospitals in the southeastern United States who had an MRI of the spine ordered in the ED. In this multi-hospital study we analyzed data from EDs at four different hospitals, which collectively received approximately 150,000 annual ED visits. All the hospitals were within one university healthcare system (none of which were trauma centers), and all EDs within these hospitals were run by emergency medicine residents and attendings. This study was approved by the institutional review board.

### Data Collection

We obtained the data from a comprehensive data warehouse. All the optimal elements of retrospective chart review were considered, based on previous studies where applicable.^22^ The inclusion criteria for this study encompassed consecutive emergent cervical, thoracic, and lumbar spine MRIs ordered between January 1, 201–December 31, 2022. The spine MRI reports were reviewed by trained personnel consisting of a medical student and research associates to classify the findings into positive, negative, or indeterminate for pathology based on the report findings and impression. Subsequently, a neuroradiologist reviewed the reports and MRI for indeterminate cases and classified these cases accordingly. The imaging findings were classified into categories based on the severity of canal stenosis or fracture: mild, moderate, severe, or no evidence of such conditions. Subsequently, positive cases were defined as those exhibiting severe stenosis and/or fracture.

**Population Health Research Capsule**

What do we already know about this issue?

*The necessity of STAT spine magnetic resonance imaging (MRI) remains a topic of debate; however, it is well-accepted that emergent spine MRIs may not always be warranted*.

What was the research question?

*What was the positivity rate of STAT spine MRI at our institution, and what are the clinical outcomes of patients following the MRI?*

What was the major finding of the study?

*The positivity rate of STAT spine MRI was 18.9%, with 3.3% admitted. Positive cases had longer ED waits (8.1 vs 7.2 hours; P < .001)*.

How does this improve population health?

*STAT spine MRI had a high negative rate, and most positive cases were discharged for non-emergent care. A better triage system may improve efficiency in the ED*.

We extracted a wide range of information from the data, including patient demographics, discharge status, any surgical interventions performed within seven days of MRI acquisition, and various time metrics such as arrival time, imaging order time, time of imaging completion, time that reports became available, and ED discharge time. Furthermore, ED chief complaint and MRI indications were recorded from reviewing the ED notes and MRI reports, respectively.

### Outcomes

We used the time points to determine time intervals: ED LOS, which was defined as the time interval between ED arrival and ED discharge; image acquisition time, which was defined as the time from image order placement to imaging completion; and image interpretation time, which was defined as the time between imaging completion and the time final report became available.

### Statistical Analysis

In addition to descriptive statistics for frequency and median with interquartile range (IQR) for time intervals, we employed Mann-Whitney U tests to compare time intervals. Chi-square test was used to compare sex differences between positive and negative spine MRI groups. Pearson correlation analysis was applied to determine the association between age and spinal MRI positivity. The significance level was set at 0.05. We conducted all statistical analyses using SPSS v29.0 (IBM Corp, Armonk, NY) and created graphs with SPSS and Prism v10.02 (GraphPad, Inc, San Diego, CA).

## RESULTS

Of the 889,527 ED encounters in the four EDs, a total of 689 spine MRIs were identified in the study period, which represented 0.1% of the total encounters. Of these, 112 (6.3%) were trauma cases including those due to falling and motor vehicle/bicycle collisions. Among the spine MRIs, 426 (61.8%) were lumbar, 234/(34.0%) were cervical, and 29 (4.2%) were thoracic MRIs. The mean age of the patients was 51.3 ±17.1 years, with females accounting for 59.5% (410/689) of the cases. Of the total, 414 were White (60.0%), 212 (30.8 %) were Black, and 9.1% (63/689) were from other racial groups.

Regarding mode of arrival, 85.0% (586/689) of patients arrived at the ED by private vehicle, 13.5% (93/689) by emergency medical services, and 1.4% (10/689) by other means. Of the total 689 patients, both trauma and non-trauma, 93.9% (647) were discharged home, 3.3% (23) were admitted for hospitalization, 1.7% (12) left against medical advice, and 1.0% (7) were discharged to other facilities. Of 23 hospitalized patients, 73.9% (17/23) underwent a surgical procedure while 26.1% (6/23) received medical management alone. Similarly, of 577 non-trauma patients, 3.5% (20) were hospitalized, and among those hospitalized, 70% (14) underwent surgery.

### Indications for STAT Magnetic Resonance imaging

The indications for STAT spine MRI, as shown in Table 1, encompassed a range of critical signs and symptoms. A majority of patients (57.5%) presented with a complaint of “back pain,” which led to hospitalization in only 3.5% of cases and necessitated surgery in 2.8% of cases. Moreover, upper extremity weakness was observed in 8.1% of patients, while lower extremity weakness was observed in 16.4% of patients. This myelopathy led to surgical intervention for 1.8% and 3.5% of these patients, respectively. Other notable indications, not presented in the table, included various concerns such as a spinal metastasis with history of malignancy, follow-up examinations guided by computed tomography (CT)/radiography findings, infectious workup in cases involving fever, patients with a history of demyelinating disease, routine annual MRI follow-ups, and instances necessitating a repeat of a previously suboptimal outpatient MRI.

### Findings on Magnetic Resonance Imaging

The overall positivity rate for spine MRIs was 18.9% (130/689), wherein 77.7% (101) of them had pure severe spinal canal stenosis, 18.5% (24) had acute vertebral fracture without spinal canal stenosis, and 3.8% (5) had acute vertebral fracture with severe spinal canal stenosis. Positivity rates varied across different regions of the spine, with thoracic MRIs showing the highest rate at 34.5% (10/29), followed by lumbar MRIs at 19.2% (82/426), and cervical MRIs at 16.2% (38/234). The positivity rate was significantly higher in the thoracic region compared to the lumbar (*P* = .04) and cervical spine (*P* = .01). Furthermore, atraumatic cases had an 18.0% (104) positivity rate, 81.7% of which (85) had pure severe spinal canal stenosis, 14.4% (15) showed acute vertebral fracture without spinal canal stenosis, and 3.8% (4) had acute vertebral fracture with severe spinal canal stenosis.

There was significant correlation between age and spine MRI positivity (r = 0.313, *P* <.001). Additionally, female gender correlated with positivity of the spinal MRI (χ^2^ = 5.076, *P* = .02), as the odds for females to have a positive spinal MRI was 0.6 (confidence interval [CI] 0.4 – 0.9). Further analysis revealed that patients’ age (after controlling for sex) significantly correlated with patient hospitalization (r = 0.08, *P* = .02, CI, 0.01 – 0.16).

**Figure 1:**
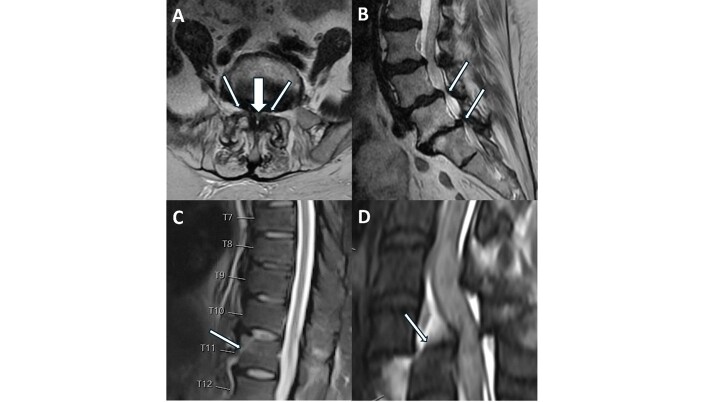
Representative positive findings in STAT magnetic resonance imaging of the spine. A: Severe spinal canal (thick arrow) and bilateral subarticular zone (thin arrows) stenosis. B: Severe spinal canal stenosis at L4–5 and L5–S1. C: Thoracic spine distraction fracture without canal stenosis. D: Cervical spine translation fracture with ligamentous and disc injury with cord edema and hemorrhage.

Other findings in the spinal MRIs included spinal cord edema or hemorrhage in 1.3% (9/689) and severe subarticular zone or lateral recess stenosis in 25.4% (175/689) of the cases (Figure 1).

### Emergency Department Wait Times

The median (IQR) image acquisition time was 2.6 (1.8 – 3.7) hours, while the median interpretation time was 1.5 (0.4 – 13.9) hours. The median LOS in the ED was 7.4 (5.7 – 9.5) hours. For non-trauma cases, median (IQR) for image acquisition, interpretation, and LOS times were 2.7 (1.8 – 3.8), 1.6 (0.5 – 14), 7.4 (5.8 – 9.5) hours, respectively. Only image acquisition time was significantly lower in trauma patients compared to non-trauma patients (*P* < 0.001).

### Emergency Departmeent Process Variability

Distribution of patients in the four EDs were 16.3% (112), 27.6% (190), 24.2% (167), and 31.9% (220). Patient characteristics and waiting times for each ED are shown in Table 2. There was a significant difference among the four EDs image acquisition time, image interpretation time, and ED LOS. MR indications for patients in each ED are presented in Supplement 1.

## DISCUSSION

This multi-hospital retrospective study demonstrated that STAT ED spine MRIs are rare, occurring in < 0.1% of ED encounters and with a low prevalence of positive findings (18.9%), which are defined as severe spinal canal stenosis and/or fracture. Among ED patients receiving spine MRI, one in 30 required hospitalizations, and one in 40 underwent surgery within seven days. Most (83.7%) non-trauma patients had hospitalization and surgery rates similar to those of the overall cohort. Furthermore, while positive MRI results led to longer ED stays, not all resulted in hospitalization or surgery. Stratifying patients by hospital revealed longer image acquisition, interpretation, and ED LOS in hospitals with a higher Black patient population.

While our study’s overall MRI positivity rate of 18.9% aligns with the range of 6.6–52% reported in previous studies for emergent spine MRIs,^7^,^23^–^28^ it is crucial to emphasize the persistent challenge of consistently low positivity rates observed across various investigations. For example, Balasubramanian et al^25^ found a positivity rate of 18.8% of 80 patients for confirmed cauda equina syndrome (CES) leading to emergency surgery. Similarly, Bell et al^26^ reported a 22% positive MRI rate for CES, and Domen et al^27^ observed a 13% positive yield in urgent MRI performed for CES. In the study by Sayed et al^29^ focusing on ED MRI for suspected epidural abscess, only 6.6% of the MRI showed positive results of 106 cases. These findings collectively underscore the persistent challenge of low positivity rates in emergent spine MRIs and emphasize the pressing need for clinical guidelines to enhance the diagnostic efficiency for spinal canal stenosis in the ED.

Interestingly, our study revealed marked differences in positivity rates across different spinal levels. The thoracic spine presented the highest positivity rate at 34.5%, while the cervical spine had the lowest at 16.2%. This variability hints at the impact of clinical decision-making, with the thoracic spine possibly prompting clinicians to order MRI with a higher pre-test probability, resulting in elevated positivity rates. Investigating the reasons for this heterogeneity in positivity by spinal level could offer valuable insights to improve overall diagnostic yield.

Building on this exploration, our study highlights “back pain or injury” as the most common (57.5%) indication for emergency spine MRI among our patients, revealing a notably lower subsequent surgery rate for back pain (2.8%) compared to that reported in the existing literature. For instance, Hussain et al^18^ found 13% (32 of 250 patients) with clinical and radiological CES underwent urgent surgery. Similarly, Kindrachuk et al^13^ recommended surgery for 12.6% of their cohort (11 of 87 patients), slightly below the previously reported rate of 15%. Moreover, Webster et al^30^ reported that 22.0% (156 patients) proceeded to surgery of the cases that had undergone MRI earlier in the course of back pain. Possible reasons for our reduced surgery incidence could be variations in patient populations, ordering behaviors, clinical indications, and the use of different diagnostic algorithms among physicians.

In a step forward we delved into the assessment of ED waiting times including ED LOS, image acquisition time, and image interpretation times to pave the way for future research on the cost effectiveness of emergent spine MRI, as well as potential resources consumed in this process. Although these time intervals are influenced by various factors, we found that the image acquisition, image interpretation, and ED LOS for these patients were 2.6, 1.5, and 7.4 hours, respectively.

Additionally, studies, such as that by Aaronson et al,^31^ highlight increased observation admissions and longer ED LOS for patients with uncomplicated back pain undergoing MRI (4.8 vs 2.7 hours). While not implying causation, these findings suggest a correlation between imaging and extended ED LOS. Gardner et al^32^ note both CT and MRI independently contribute to approximately 36 minutes additional time. Our longer ED LOS may result from variations in study design and differences in target patients and settings. Strategies to reduce ED LOS include enhancing the management of patients awaiting MRI results, potentially involving measures such as transient hospitalization during the waiting period. Current policies mandating waiting for the final MRI spine report before patient transfer could be revisited to expedite patient flow without compromising care quality.

## LIMITATIONS

Limitations in our retrospective study include potential missing or inaccurate data, inherent to this study design. Classification bias may occur in categorizing indeterminate results, relying on subjective judgment. The study lacks an assessment of imaging exam quality, and variations in recording ED time metrics across sites could have introduced inconsistency. Additionally, excluding patients seeking care from hospitals outside university health system, being performed in a single university system in a specific metropolitan area, and absence of a Level I trauma center may impact the generalizability of the findings. Furthermore, our study specifically examines canal stenosis and fractures as indirect indicators for potential surgery candidates. However, we acknowledge that other emergent medical conditions, while not surgical in nature, such as transverse myelitis and osteomyelitis without epidural abscess, may have been overlooked in our positive cases. Additionally, we did not include positive cord signal as an independent factor to identify positive MRI. Finally, our reliance on admission and surgery as clinical interventions may overlook other ED interventions.

Given the low positive findings rate and the predominance of discharged patients, future research should delve into risk stratification and alternative care pathways beyond the ED for this patient population. Additionally, a more standardized checklist to define the positivity of STAT spine MRI in the ED perhaps should be explored in future studies.

## CONCLUSION

Our study revealed the incidence of positive emergent spine MRI, with a relatively small number necessitating hospitalization or surgery. This suggests a potential overuse of ED spine MRI. Selecting patients for emergency spinal MRIs presents a challenge due to the wide spectrum of diseases and non-specific clinical presentations. Our study also highlights heterogeneity in positivity rates based on the imaged spinal level, with the thoracic spine demonstrating the highest positivity. Interestingly, the implementation of diagnostic algorithms has shown potential in increasing positivity rates, and hence reducing time, healthcare expenses, and improving patient outcomes. Further research is needed to optimize the triage process for patients requiring emergent spine MRI.

## Supplementary Information



## Figures and Tables

**Table 1:** Indications for STAT magnetic resonance imaging in the Emergency Department.

MRI Indication as ordered	Presentation	Hospitalization	Surgery
Back pain/injury	57.5% (396/689)	3.5% (14/396)	2.8% (11/396)
Lower extremity pain	25.7% (177/689)	2.2% (4/177)	1.1% (2/177)
Numbness	20.7% (143/689)	2.8% (4/143)	1.4% (2/143)
Neck pain/injury	16.4% (113/689)	0.9% (1/113)	0.9% (1/113)
Fall	9.7% (67/689)	3.0% (2/67)	3.0% (2/67)
Motor vehicle / bicycle accident	6.5% (45/689)	2.2% (1/45)	2.2% (1/45)
Upper extremity pain	5.9% (41/689)	2.4% (1/41)	0%
Headache / head injury	3.3% (23/689)	0%	0%
Post-surgery/spinal injection complications	16.0% (110/689)	5.4% (6/110)	2.7% (3/110)
Fecal incontinence	5.7% (39/689)	5.1% (2/39)	5.1% (2/39)
Constipation	2.2% (15/689)	6.7% (1/15)	6.7% (1/15)
Urinary incontinence	10.3% (71/689)	2.8% (2/71)	1.4% (1/71)
Urinary retention	4.9% (34/689)	2.9% (1/34)	2.9% (1/34)
Upper extremity weakness	8.1% (56/689)	3.6% (2/56)	1.8% (1/56)
Lower extremity weakness	16.4% (113/689)	6.2% (7/113)	3.5% (4/113)
Saddle anesthesia	3.9% (27/689)	3.7% (1/27)	3.7% (1/27)
Hyperreflexia / spasm	0.6% (4/689)	0%	0%
Paresthesia	20.3% (140/689)	1.4% (2/140)	1.4% (2/140)
Total	100% (689/689)	3.3% (23/689)	2.0% (14/689)

* Some patients experienced more than one indication for hospitalization and surgery.

*MRI*, magnetic resonance imaging.

**Table 2:** Differences in emergency department patient populations and wait times.

	ED 1 (n = 112)	ED 2 (n = 190)	ED 3 (n = 167)	ED 4 (n = 220)	P-value
Age mean (SD) yrs	48.6 (14.7)	48.4 (17)	50.1 (15.5)	56.3 (18.6)	<.001
Female sex (n)	54.5% (61)	61.1% (116)	59.3% (99)	60.9% (134)	.67
Race (Black [n] / White [n])	59.8 % [67] / 36.6% [41]	14.7% [27] / 71.6% [136]	43.1% [72] / 52.1% [87]	20.5% [45] / 68.2% [150]	<.001
Trauma (n)	17% (19)	20.5% (39)	11.4% (19)	15.9% (35)	.13
Hospitalization rate	2.7% (3)	5.8% (11)	3% (5)	1.8% (4)	.14
Surgery	1.8% (2)	3.7% (7)	3% (5)	1.4% (3)	.44
MRI positivity rate	20.5% (23)	16.8% (32)	18.6% (31)	20% (44)	.82
Median (IQR) image acquisition time [hours]	2.7 (1.8 – 4.3)	2.5 (1.7 – 3.4)	3.1 (2.1 – 4.1)	2.3 (1.5 – 3.1)	<.001
Median (IQR) image interpretation time	8.5 (1.2 – 16.9)	0.9 (0.4 – 6.8)	14.2 (3.5 – 18.0)	0.6 (0.3 – 1.6)	<.001
Median (IQR) ED LOS	8.6 (6.7 – 11.1)	6.5 (5.1 – 8.1)	9.0 (7.5 – 11.4)	6.6 (5.5 – 8.1)	<.001

*ED*, emergency department; *SD*, standard deviation; *MRI, magnetic resonance imaging*; *IQR*, interquartile range; *LOS*, length of stay.
