# Supplementary material for: Utility of Emergent Spine MRI in the Emergency Department
Source: West J Emerg Med. 2025 Jul 12;26(4):936–42. doi: 10.5811/westjem.32802 (PMC12342433; doi:10.5811/westjem.32802)
Supplement: Supplementary file 1 [file wjem-26-936-s001.docx]

**Supplement 1.** Magnetic resonance imaging indications in each emergency department.

|  | MRI indications | **ED 1 (n = 112)** | **ED 2**  **(n = 190)** | **ED 3**  **(n = 167)** | **ED 4**  **(n = 220)** |
| --- | --- | --- | --- | --- | --- |
|  | Back pain/injury | 62.5% (70) | 55.8% (106) | 64.7% (108) | 50.9 (112) |
|  | Lower extremity pain | 16.1% (18) | 25.3% (48) | 21% (35) | 34.1% (75) |
|  | Numbness | 15.2% (17) | 22.6% (43) | 24% (40) | 19.5% (43) |
|  | Neck pain/injury | 21.4% (24) | 22.1% (42) | 7.2% (12) | 15.5% (34) |
|  | Fall | 8.9% (10) | 13.2% (25) | 6.6% (11) | 9.5% (21) |
|  | Motor vehicle / bicycle accident | 8% (9) | 7.4% (14) | 4.8% (14) | 6.4% (14) |
|  | Upper extremity pain | 5.4% (6) | 6.8% (13) | 4.2% (7) | 6.8% (15) |
|  | Headache / head injury | 1.8% (2) | 5.3% (10) | 3% (5) | 2.7% (6) |
|  | Post-surgery/spinal injection complications | 23.2% (26) | 13.2% (25) | 10.8% (18) | 18.2% (40) |
|  | Fecal incontinence | 7.1% (8) | 4.2% (8) | 9% (15) | 3.6% (8) |
|  | Constipation | 3.6% (4) | 1.6% (3) | 2.4% (4) | 1.8% (4) |
|  | Urinary incontinence | 13.4% (15) | 7.9% (15) | 13.8% (23) | 8.2% (18) |
|  | Urinary retention | 7.1% (8) | 4.2% (8) | 6.6% (11) | 3.2% (7) |
|  | Upper extremity weakness | 10.7% (12) | 6.8% (13) | 7.2% (12) | 8.2% (18) |
|  | Lower extremity weakness | 15.2% (17) | 14.2% (27) | 22.2% (37) | 14.1% (31) |
|  | Saddle anesthesia | 2.7% (3) | 4.2% (8) | 5.4% (9) | 3.2% (7) |
|  | Hyper-reflexia / spasm | 0 | 0.5% (1) | 1.2% (2) | 0.5% (1) |
|  | Paresthesia | 18.8% (21) | 21.6% (41) | 19.2% (32) | 20.9% (46) |

*ED*, emergency department; *MRI,* magnetic resonance imaging.
